# Relationship between physical activity and depressive symptoms in older Korean adults: moderation analysis of muscular strength

**DOI:** 10.1186/s12877-022-03610-6

**Published:** 2022-11-21

**Authors:** Ji-Young Kong, Haeryun Hong, Hyunsik Kang

**Affiliations:** College of Sport Science, Sungkyunkwan University, Suwon, 16419 Republic of Korea

**Keywords:** Late-life depression, Physical activity, Muscular fitness, Older adults

## Abstract

**Background:**

This population-based cross-sectional study examined the associations between physical activity (PA) and lower body muscle strength (LBMS) with late-life depression in a representative sample of older Korean adults aged 65 years and older.

**Methods:**

The data used in the current study (*n* = 10,097/60% women) were extracted from the 2020 Korea Longitudinal Study on Aging, which is a nationwide population-based survey conducted in Korea. Depressive symptoms were assessed with the Geriatric Depression Scale Short-Form. PA and LBMS were evaluated with a self-reported questionnaire and the 5 times sit-to-stand test, respectively. Covariates include age, gender, body mass index, education level, smoking status, alcohol intake, and comorbidity.

**Results:**

Insufficient PA had higher odds of depression (odds ratio [OR] = 1.201, 95% confidence interval [CI] = 1.035–1.393, *p* = 0.016), even after adjustments for all covariates, compared to sufficient PA. Poor LBMS had higher odds of depression (OR = 2.173, 95% CI = 1.821–2.593, *p* < 0.001), even after adjustments for all covariates, compared to good LBMS. Particularly, a significant moderation effect of LBMS on the relationship between PA and depressive symptoms was observed (β = 0.3514 and 95% CI = 0.1294 ~ 0.5733, *p* < 0.001). Individuals with poor LBMS had a greater odd of depression associated with physical inactivity compared to their counterparts with good LBMS.

**Conclusions:**

The results of this study support the importance of promoting muscular strength through regular exercise as a preventive strategy against late-life depression in Korean adults.

## Background

Late-life depression (LLD) is defined as a mental illness occurring for the first time at the age of 60 years or older, and its prevalence ranges from 7.7 to 81.1% depending on ethnicity [1]. LLD has become one of the leading causes of disability in the world, becoming a major contributor to the global burden of disease (https://www.who.int/news-room/fact-sheets/detail/depression). Etiologically, LLD correlates with cognitive impairments and increased odds of dementia and mortality [2]. According to the South Korea National Health Insurance Service-Senior cohort for 2002–2013, the prevalence rates of depression were highest in women at the ages of 65–79 years and in men at the ages of 75–84 years, putting these age groups at major risk for suicide [3].

Despite its high prevalence and clinical significance, however, LLD is underrecognized, undertreated, and often viewed as a normal part of the aging process [4]. Pharmacotherapy and psychotherapy are efficacious for reducing depressive symptoms in older subjects [5], but they can be expensive for healthcare systems [6]. Furthermore, the side effects of antidepressants in older subjects, including falls, cardiovascular events, fractures, epilepsy, hyponatremia, and increased risk of all-cause mortality, are common [7] and have impacts on treatment outcomes [8]. Thus, it is essential to identify effective alternative options.

PA is defined as any movement of skeletal muscles that results in energy expenditure over the resting metabolic rate [9] Muscular strength, which is a component of muscular fitness, refers to the maximal amount of force a muscle group can produce in a single effort. Although muscular strength is largely inherited, it is also somewhat influenced by environmental factors such as PA and nutrition [10].

The preventive and therapeutic potentials of PA are recognized in patients with depression, and PA is recommended as a non-pharmacologic alternative against LLD [8]. Likewise, measures of upper and lower body muscle strength are associated with a lower risk of depression and/or depressive symptoms in older populations [11–13]. In our previous study involving older Korean adults, we showed that muscle mass and muscle function are inversely associated with depressive symptoms [14]. The inverse association between those healthy behaviors and depressive symptoms is reviewed and well summarized in a systematic review and meta-analysis involving 26 studies involving 87,508 adults from 26 countries [15].

PA and muscle strength, as two positive contributors to mental health, are often interrelated to each other such that some individuals having sufficient daily PA may have adequate muscle strength too, and vice versa, suggesting the importance of taking both into account when assessing the risks for LLD. Nevertheless, the associations between PA and muscle strength with LLD in older adults are unclear. This study aimed to examine the moderation effect of lower body muscle strength (LBMS) on the relationship between PA and depression symptoms in a representative sample of older Korean adults.

## Methods

### Data source and study participants

**Fig. 1:**
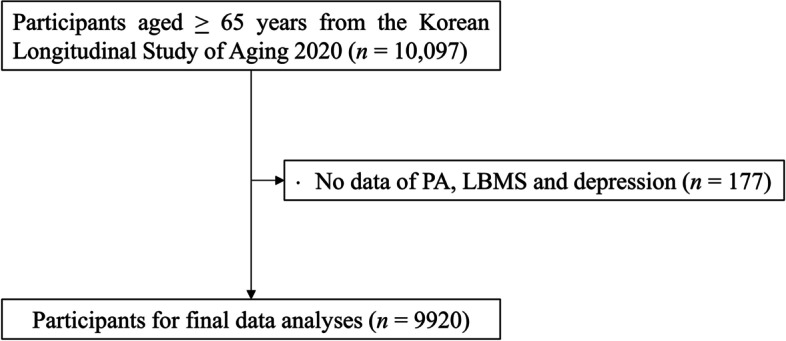
Flow chart for the selection of study participants. PA: physical activity; LBMS: lower body muscle strength

The data for the current study were extracted from the 2020 Korean Longitudinal Study on Aging (KLoSA), which is a nationwide population-based survey biannually conducted in Korea since 2006 (wave 1). As shown in Fig. 1, a total of 10,097 adults aged 65 years and older (6,062 females/60.0%) participated in the 2020 survey (wave 8). Respondents with no information in terms of the Korean version of the Geriatric Depression Scale Short-Form (K-GDS-SF), PA, and LBMS data (*n* = 177) were excluded. The remaining 9,920 individuals (4,035 males and 6,062 females) were used for final data analyses. Detailed information regarding the KLoSA is available through the national public database (https://survey.keis.or.kr/eng/myinfo/login.jsp).

### Measured variables

#### Assessment of depressive symptoms

Depressive symptoms were assessed using the K-GDS-SF. Depression was defined as a score ≥ 8 on the K-GDS-SF or physician-diagnosed depression or taking anti-depressant medication(s). A cutoff score of 8 for mental illness screening was previously assessed and validated in older Korean adults [16].

#### Physical activity and lower body muscle strength

PA was assessed by asking whether the subjects participated in any type of exercise at least once a week, and the frequency and duration of exercise were recorded [17]. The volume of PA (minutes per week) was calculated by multiplying duration and frequency, and it was categorized as sufficient (150 min per week) or insufficient (< 150 min per week) according to the global recommendations for PA (https://www.who.int/publications/i/item/9789241599979).

A test known as the 5 times sit-to-stand test (5STST) was used to evaluate LBMS according to the protocols described previously [18]. In brief, participants were instructed to stand from a sitting position on a chair with both arms folded across the chest 5 times as fast as possible. Performance on 5 times sit-to-stand tests is measured with scores for completeness (1 = completed successfully, 2 = tried but failed to complete, 3 = could not perform at all). For purposes of analysis herein, “completed successfully” was categorized as good, while “tried but failed to complete” and “could not perform at all” were collapsed and categorized as poor. The validity and reliability of the 5STST for the assessment of LBMS were previously tested and reported in Korean elderly persons [19] and others [20].

#### Covariates

The covariates included in this study were age (years), gender (male or female), body mass index (BMI), educational level (elementary or lower, middle/high school, college or higher), smoking status (current/past smoker or non-smoker), alcohol intake (0, 1–6, ≥ 7 times/week), and comorbidity. Comorbidity was determined using diagnoses of at least one of 16 selected chronic conditions previously reported by a doctor [21].

### Statistical analyses

**Fig. 2:**
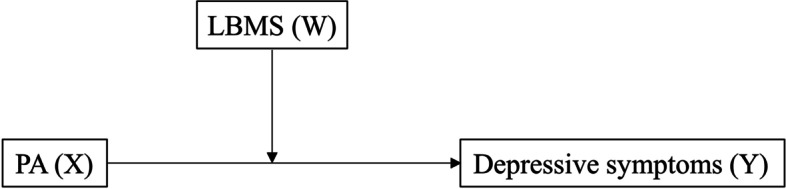
Conceptual diagram of the relationship between physical activity (PA, X) and depressive symptoms (Y) moderated by lower body muscle strength (LBMS, W)

Data distribution normality and multicollinearity were verified using quantile–quantile plots and variance of inflation factors, respectively. Student’s *t*-tests and chi-square tests were used to compare continuous and categorical variables, respectively, between Individuals with and without depression. Linear regression was used to determine the relationships between measured parameters and depressive symptoms. Multivariate logistic regression was used to estimate odds ratios (ORs) and 95% confidence intervals (CIs) of depression according to PA and LBMS. Finally, as illustrated in Fig. 2, a moderation analysis of LBMS (moderator, W) on the relationship between PA (continuous, X) and depressive symptoms (continuous, Y) was conducted using PROCESS macro by Andrew Hayes [22]. The statistical significance of the model was assessed with bias-corrected bootstrapping (*n* = 10,000) and 95% CIs. The statistical significance of a relationship was evaluated with a non-zero value of a 95% bootstrapped CI. All other statistical significances were evaluated at *α* = 0.05 using SPSS version 27.0 for Windows (IBM Corporation, Armonk, NY, USA).

## Results

Table 1 describes the physical and demographic characteristics of study participants by depression status. Individuals with depression were older (*p* < 0.001), likely to be married and living without a spouse (*p* < 0.001), and have lower BMI values (*p* < 0.001), fewer years of education (*p* < 0.001), higher multi-morbidity (*p* < 0.001), insufficient PA, and poor LBMS (*p* < 0.001) in comparison to individuals without depression.

**Table 1:** Descriptive statistics of study participants

Variables	Not depressed (*n* = 9042/89.6%)	Depressed (*n* = 878/8.7%)	Total (*n* = 9920/100%)	*p-*value
Mean age, (years)	73.3 ± 6.5	74.7 ± 7.0	73.4 ± 6.5	< 0.001
Body mass index (kg/m^2^)	23.6 ± 2.6	23.1 ± 2.7	23.6 ± 2.6	< 0.001
Gender, n (%)				0.013
Male	3654 (40.4)	317 (36.1)	3971 (40.0)	
Female	5388 (59.6)	561 (63.9)	5949 (60.0)	
Marriage status				< 0.001
Never married	35 (0.4)	6 (0.7)	41 (0.4)	
Married with spouse	5443 (60.2)	406 (46.2)	5849 (59.0)	
Married without spouse	3564 (39.4)	466 (53.1)	4030 (40.6)	
Educational background, n (%)				< 0.001
Elementary or less	3969 (43.9)	463 (52.7)	4431 (44.7)	
Middle/high school	4590 (50.8)	394 (44.9)	4984 (50.2)	
College or higher	484 (5.4)	21 (2.4)	505 (5.1)	
Smoking status, n (%)				0.154
Never smoked	8062 (89.2)	769 (87.6)	8831 (89.0)	
Current/past smokers	980 (10.8)	109 (12.4)	1089(11.0)	
Alcohol intake (times/week)				0.212
0	7783 (86.1)	740 (84.3)	8523 (85.9)	
1–6	1159 (12.8)	129 (14.7)	1288 (13.0)	
> 7	100 (1.1)	9 (1.0)	109 (1.1)	
Multi-morbidity, n (%)				< 0.001
None	1598 (20.7)	80 (10.7)	1678 (19.8)	
Single	2678 (34.7)	244 (32.8)	2922 (34.5)	
Multiple	3451 (44.7)	421 (56.5)	3872 (45.7)	
Physical activity, n (%)				< 0.001
Sufficient	4802 (53.1)	385 (43.8)	5187 (52.3)	
Insufficient	4240 (46.9)	493 (56.2)	4733 (47.7)	
Lower body muscle strength, n (%)				< 0.001
Good	6781 (78.5)	504 (61.0)	7285 (77.0)	
Poor	1853 (21.5)	322 (39.0)	2175 (23.0)	

Table 2 represents bivariate correlations between depressive symptoms and the measured parameters of study participants. Depressive symptoms were shown to be significantly correlated with age (β = -0.113 and *p* < 0.001), marital status (β = -0.290 and *p* = 0.014), education (β = 0.863 and *p* < 0.001), smoking status (β = -0.551 and *p* = 0.002), PA (β = -0.444 and *p* < 0.001), and LBMS (β = -2.320 and *p* < 0.001).

**Table 2:** Linear regression for determinants of depressive symptoms

Variables	beta	95% CI	r^2^_part_	*p* value	VIF
Age	-0.113	-0.133 ~ -0.092	-0.108	< 0.001	1.651
Gender	0.162	-0.093 ~ 0.417	0.013	0.212	1.492
Body mass index	0.023	-0.019 ~ 0.064	0.011	0.283	1.024
Marriage	-0.290	-0.521 ~ -0.059	-0.025	0.014	1.237
Education	0.863	0.761 ~ 0.964	0.167	< 0.001	1.483
Smoking	-0.551	-0.906 ~ -0.196	-0.031	0.002	1.198
Alcohol intake	0.269	-0.014 ~ 0.552	0.019	0.062	1.191
Multi-comorbidity	-0.058	-0.198 ~ 0.082	-0.008	0.417	1.094
Physical activity	-0.444	-0.655 ~ -0.233	-0.041	< 0.001	1.047
Muscle strength	-2.320	-2.611 ~ -2.029	-0.157	< 0.001	1.339

*CI* Confidence interval, *VIF* Variance inflation factor, *PA* Physical activity, *MS* Muscle strength

Table 3 represents the ORs and 95% CIs of depression according to PA and LBMS-based subgroups. Individuals with insufficient PA were at increased odds of depression (OR = 1.450 and 95% CI = 1.261 ~ 1.667, *p* < 0.001) compared to individuals with sufficient PA. The increased OR for depression remained significant (OR = 1.201 and 95% CI = 1.035 ~ 1.393, *p* = 0.016) even after adjustments for age, marital status, education level, smoking status, physical activity, and LBMS. Likewise, individuals with poor LBMS were at increased odds of depression (OR = 2.338 and 95% CI = 2.014 ~ 2.714, *p* < 0.001) compared to individuals with good LBMS. The increased OR for depression remained significant (OR = 2.173 and 95% CI = 1.821 ~ 2.593, *p* < 0.001) even after adjustments for all the covariates.

**Table 3:** Odds ratios (ORs) and 95% confidence intervals (CIs) of depression by physical activity and muscle strength

Predictors		Model 1		Model 2	
		OR (95% CI)	*p* value	OR (95% CI)	*p* value
PA					
Sufficient	1 (reference)		< 0.001	1 (reference)	
Insufficient	1.450 (1.261 ~ 1.667)			1.201 (1.035 ~ 1.393)	0.016
LBMS					
Good	1 (reference)			1 (reference)	
Poor	2.338 (2.014 ~ 2.714)		< 0.001	2.173 (1.821 ~ 2.593)	< 0.001

*PA* Physical activity, *LBMS* Lower body muscle strength

Model 1: unadjusted

Model 2: adjusted for age, gender, marriage, education, smoking, physical activity (for LBMS), and lower body muscle strength (for PA)

Table 4 shows the relationship between PA (X) and depression (Y) by LBMS (W). A significant moderation effect of LBMS on the relationship between PA and depression was observed (β = 0.3407 and 95% CI = 0.1190 ~ 0.5625). The moderating effect of PA remained statistically significant (β = 0.3514 and 95% CI = 0.1294 ~ 0.5733), even after adjustments for all covariates.

**Table 4:** Moderation analysis of physical activity for the relationship between muscle strength and depressive symptoms

Predictors	Coefficients		SE		t		*p*		95% CI		
									Lower		Upper
Model 1 (R^2^ = 0.0037, F = 11.7336, *p* < 0.001)											
Physical activity	-0.1094		0.0802	-2.0546		0.0399		-0.2138		-0.0050	
Muscle strength	-0.2553		0.0533	-1.3600		0.1739		-0.6233		0.1127	
Interaction	0.3407		0.1877	3.0123		0.0026		0.1190		0.5625	
R^2^ change due to the moderator = 0.0010 (F = 9.0740, *p* = 0.0026)											
Model 2 ( R^2^ = 0.0060, F = 8.1013, *p* < 0.001)											
Muscle strength	-0.1061	0.0535		-1.9816		0.0476		-0.2111		-0.0011	
Physical activity	-0.1981	0.1888		-1.0493		0.2941		-0.5681		0.1720	
Interaction	0.3514	0.1132		3.1033		0.0019		0.1294		0.5733	
R^2^ change due to the moderator = 0.0010 (F = 9.6303, *p* = 0.0019)											

*SE* Standard error, *CI* Confidence interval

Model 1 unadjusted

Model 2 adjusted for age, marriage, education, and smoking

**Fig. 3:**
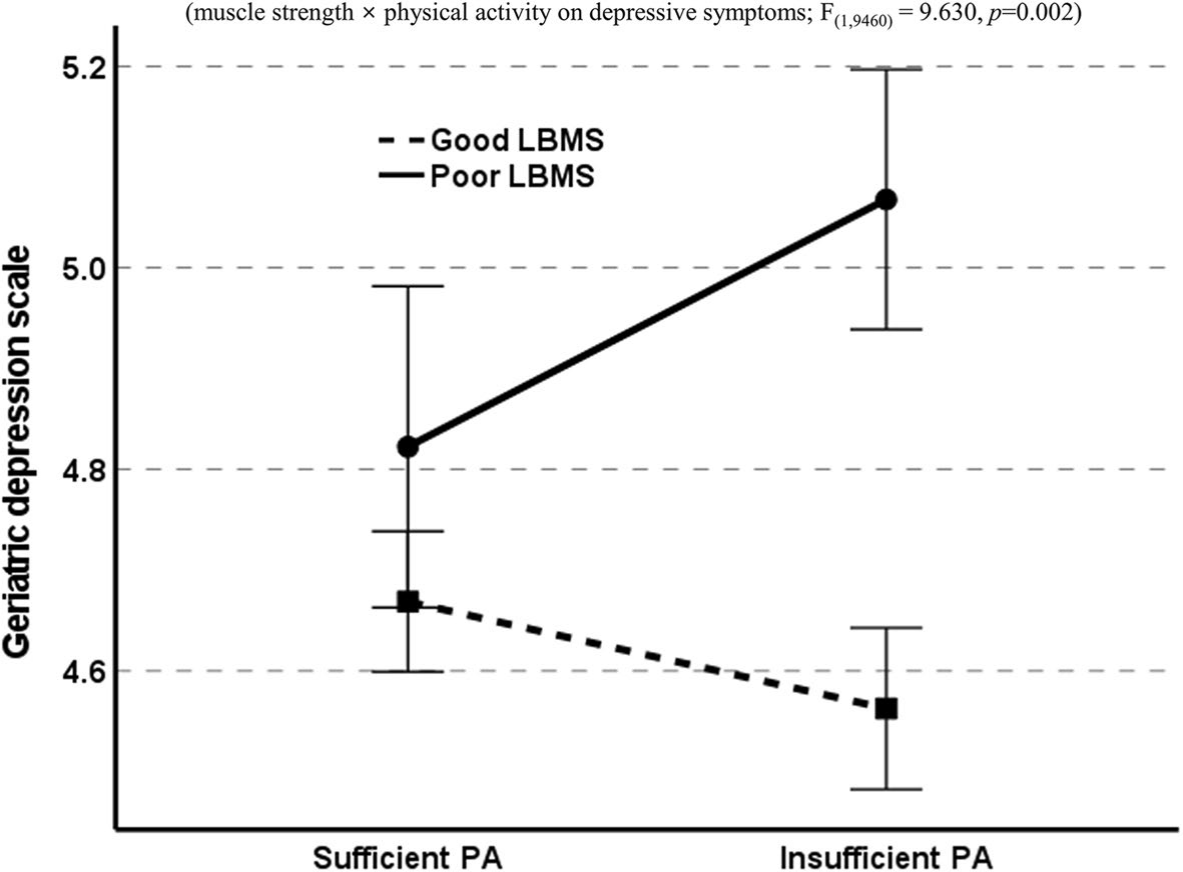
Effect of lower body muscle strength (LBMS) on the relationship between physical activity (PA) and depressive symptoms in Korean older adults

The interaction was further investigated to better understand the moderation effect of LBMS on the relationship between PA and depressive symptoms. As shown in Fig. 3, insufficient PA had a greater impact on depressive symptoms in individuals with poor LBMS (F_(1,9460)_ = 9.630, *p* = 0.002) compared to the impact of insufficient PA on depressive symptoms in individuals with good LBMS.

## Discussion

This population-based study examines PA and LBMS in relation to depression in older Korean adults, reporting an inverse relationship between PA and LBMS with LLD. In particular, the study suggests that individuals with poor LBMS are likely to have greater odds of LLD associated with insufficient PA than individuals with good LBMS.

The current findings are consistent with the findings of previous studies investigating the inverse association between PA and depressive symptoms and/or depression in older populations from different countries [23–25]. In a four-year follow-up study involving 32,392 middle-aged and older adults from 14 European countries, Marques et al. [26] investigate the relationship between PA and depressive symptoms to show that moderate and vigorous levels of PA were inversely related to depression and/or depressive symptoms at baseline and lower depression scores after four years. By conducting a systematic review and meta-analysis involving more than two million person-years from 15 prospective studies, the work of Pearce et al. [27] examines the association between daily PA and incident depression and finds that individuals who met the recommended weekly PA had a lower risk of depression in comparison to their counterparts with no PA.

Our current findings are also consistent with the findings of previous studies reporting an inverse association between LBMS and LLD in Western and Asian older adults. By analyzing data obtained from the National Health and Nutrition Evaluation Survey (NHANES, 1999–2006), Cangin et al. [28] show that aerobic PA and muscle-strengthening PA were significantly associated with a lower risk of depression in US men and women. Furthermore, the antidepressant effects of muscle-strengthening PA were shown to be independent of aerobic PA. In a seven-year follow-up study involving 228 middle-aged and older adults without depression at baseline, Bao et al. [29] examine the association between handgrip strength and the 5 times sit-to-stand test (5STST) and incident depression. That study shows that greater handgrip strength at baseline was associated with a lower seven-year incident depression, while poor 5STST at baseline was an independent predictor of seven-year incident depression. These findings demonstrate the importance of measuring both upper-body muscle strength and LBMS in assessing the risk for depression. The work of Galán-Arroyo et al. [30] examines the association between the 30 s sit-to-stand test and the geriatric depression scale (GDS) in 685 elderly women with depression and shows an inverse relationship between LBMS and six of 15 items on the GDS. Likewise, upper-body muscle strength is also shown to be associated with depression in older adults [31–33].

Several mechanisms may explain the antidepressant effects of PA and LBMS in sample populations of adults. First, PA reduces depressive symptoms via stimulation of neuroplasticity implicated in depression [34], attenuation of inflammation [35], enhanced resilience to oxidative and physiological stress [36], and promotion of self-esteem, social support, and self-efficacy [25]. Second, muscle strength positively contributes to PA and exercise habits [37] and physical functioning [38]. Therefore, individuals with good LBMS are less likely to suffer from depression in comparison to individuals with poor LBMS. Third, muscle strength is associated with a lower risk of geriatric health conditions, such as sarcopenia [39] and functional limitations and disabilities [40], which are important risk factors for late-life depression. Accordingly, individuals with good LBMS are likely to be not frail or robust and less dependent and have fewer difficulties in performing activities of daily living [41]. Collectively, this set of qualities provides protection against depression [42, 43]. Fourth, the pathologic mechanisms of depression involve cell death, disrupted neurogenesis, neuroinflammation, and endoplasmic reticulum stress [44]. On the other hand, the contraction-induced release of cytokines and myokines into circulatory systems may provide beneficial effects on depression via these regulatory mechanisms [45].

In particular, ours is the first study to report the moderation effect of LBMS on the relationship between PA and depression, which can be explained through several mechanisms. First, the moderation effect of LBMS on the relationship between PA and depression may be explained via sarcopenia and its relationship to depression. Insufficient PA may lead to the loss of muscle mass and strength [46], resulting in an increase in the risk for depression. Consequently, good LBMS may attenuate the impact of insufficient PA on the risk for dynapenia and depression. Second, muscle strength may attenuate the impact of insufficient PA on depression via fewer disabilities, fewer functional limitations, more independence, and a better quality of life. Third, muscle strength is positively associated with self-esteem, social support, and self-efficacy, each of which may negate the impact of insufficient PA on depression. Lastly, muscle contraction-induced myokines and/or anti-inflammatory cytokines may attenuate the impact of insufficient PA on the pathophysiology of depression via the regulator mechanisms discussed above [44].

This study has limitations. First, the cross-sectional nature of the study does not allow any cause-and-effect explanation. Second, although LBMS is a reliable index of overall muscle strength [47] and is strongly associated with handgrip strength in community-dwelling older adults [48], handgrip strength is the most frequently used measurement of muscle strength, especially in geriatric populations [49]. Thus, considering both upper and lower body muscle strength may improve the predictor role of muscular strength in relation to depression or incident depression. Third, we cannot completely rule out the chance of measurement errors in the self-reported PA questionnaire due to its inherited limitations [50].

## Conclusion

In summary, this population-based cross-sectional study examines the associations between PA, muscle strength, and depression in older Korean adults. The current findings show that LBMS is an important moderator in determining the relationship between PA and depressive symptoms, suggesting the need for an intervention to promote LBMS as well as the need to verify the present findings in longitudinal research. 

## Data Availability

The datasets used and/or analysed during the current study are available from the corresponding author on reasonable request.
